# ANCA-Associated Vasculitides and Hematologic Malignancies: Lessons from the Past and Future Perspectives

**DOI:** 10.1155/2019/1732175

**Published:** 2019-05-06

**Authors:** Marco Folci, Giacomo Ramponi, Dana Shiffer, Aurora Zumbo, Michele Agosti, Enrico Brunetta

**Affiliations:** ^1^Department of Biomedical Sciences, Humanitas University, Milan, Italy; ^2^Internal Medicine, Humanitas Research Hospital, Milan, Italy; ^3^University of Milan, Milan, Italy; ^4^Rheumatology Unit, ASST Fatebenefratelli Sacco, Milan, Italy

## Abstract

The purpose of this paper is to collect and summarize all evidences relating to an association between ANCA-associated vasculitides (AAVs) and hematologic malignancies, in the form of either a paraneoplastic vasculitis or leukemias and lymphomas developing on a preexisting vasculitis. Additionally, the role of cyclophosphamide in vasculitis treatment has been assessed and compared to rituximab. Paraneoplastic AAV seems to be an uncommon presentation of hemopathies. Hematologic malignancy risk in AAV is more likely to be increased by cyclophosphamide, although not yet definitely proven. Furthermore, the pathogenesis of ANCA-associated vasculitis has been reviewed with particular emphasis on the role of proteinase 3 (PR3) in fuelling granulomatosis with polyangiitis (GPA) inflammation. PR3 is a bactericidal protein expressed by neutrophilic granules and on their plasma membrane. Derangements in its expression and function have been linked to leukemias and GPA alike. PR3-derived PR1 peptide is being studied as an immunotherapy target in leukemia and multiple myeloma. This study is aimed at bringing together various evidences from the field of immunological and hematological research, at exposing contradictions, and at revealing novel insights on the association between ANCA-associated vasculitis and hematologic malignancies.

## 1. Introduction

The preservation of self-antigens from misdirected immune responses is allowed by multiple central and peripheral control mechanisms, which constitute the basis of immune tolerance. When a defect in these processes occurs, the survival and proliferation of autoreactive cellular clones occur in what is generally defined as tolerance breakdown [1]. As a result, a vicious cycle of immune dysregulation and tissue inflammation leads to various autoimmune diseases, among which vasculitides are probably the most complex in terms of organ involvement, plurality of presentations, and severity of tissue damage.

Similarly, the expansion of neoplastic clones is normally impeded by immune-mediated surveillance mechanisms. When these fail, usually due to a complex evasion strategy orchestrated by the growing neoplasia, the capacity to detect and clear cancer cells becomes strongly impaired. This condition leads to expansion of the tumour which finds space to invade surrounding tissues, recruit immune cells to foster its growth, and diffuse through the bloodstream. This feature is of such importance that it has been proposed as one of the hallmarks of carcinogenesis process [2]; in fact, several of the most promising new drugs for solid tumours have been developed based on this knowledge [3]. The safety and efficacy of many of these molecules have already been established in various neoplastic diseases, solid and hematological alike [4]. Nevertheless, treatment is still limited in most cases with ongoing research striving to narrow the gap. This is especially the case for many hematologic malignancies (HM) [5], as demonstrated by a promising target of immune-mediated antitumour responses: PR1, a nine-amino acid-long HLA-A2-restricted peptide, which derives from proteinase 3 (PR3). Recently, a vaccine was derived from the peptide and proved to be safe as well as clinically effective against myeloid malignancies in a phase I/II clinical trial [6]. Following a different approach, TCR-like antibodies with high PR1 affinity were developed and their activity was studied in vitro. These were able to selectively target acute myeloid leukemia (AML) [7] progenitor cells while leaving normal colony-forming units (CFUs) from healthy donors unharmed [8].

Interestingly, PR3 long owed its fame for being the putative autoantigen in granulomatosis with polyangiitis (GPA), previously named Wegener's granulomatosis. GPA belongs to the group of ANCA-associated vasculitides (AAVs), together with microscopic polyangiitis (MPA) and eosinophilic granulomatosis with polyangiitis (EGPA), also known as Churg-Strauss syndrome. PR3 autoantibodies, cANCA, are strongly associated with GPA and MPA, although their presence has been reported in several conditions such as inflammatory bowel diseases [9]. Even though the implication of PR3 in GPA and MPA has been disputed until recently [10], emerging studies support the fundamental role of this molecule in the pathogenesis of the disease [11]. The ominous and apparently contradictory role of PR3 in GPA and myeloid malignancies, in which it respectively seems to propel and suppress the immune response, will be reviewed exploring all known associations between the groups of AAV and HM [12].

## 2. Paraneoplastic AAV: When Lymphoproliferative Disorders Trigger Autoimmunity

AVVs have long been known to be associated with solid cancers, in particular kidney and colon cancer [13]. Moreover, several case reports have been published documenting a concurrent development of AAV and HM. Hamidou et al. described a 56-year-old man presenting with prolonged fever, weight loss, multiple lymphadenopathies, cutaneous purpura, and mononeuritis multiplex who was found to have high cANCA titres [14]. A systemic vasculitis involving the skin, the lungs, and the kidneys was diagnosed on a clinical base; however, lymph node examination provided evidence of concurrent T cell lymphoma [14]. A different research group shared the case of a 77-year-old man sent to the ED by his primary care physician after finding an elevated serum creatinine (from 2.2 mg/dl to 4.5 mg/dl in one week). On admission, he complained of persistent fatigue and lack of appetite for several months. He had positive PR3-ANCA. A kidney biopsy revealed the coexistence of a renal-infiltrating mantle cell lymphoma and a pauci-immune glomerulonephritis [15]. Several other case reports have described an AAV likely triggered by an underlying hematopoietic malignancy [16–18]. An association between the developments of AAV in patients with malignancies was also reported in a case series which included all types of malignancy [19]. A retrospective multicenter study described a total of 16 patients having both AAV and HM, among which 9 had a time interval of less than three months between the two diagnoses. The main associated malignancies were non-Hodgkin's lymphoma which was found in 7 patients and myelodysplasias which were present in 5 patients. Hodgkin's lymphoma, multiple myeloma, and other blood neoplasms cover the rest of the dataset [20]. The authors report that the five cases of myelodysplastic syndromes associated with AAVs were mostly refractory anemia with excess blasts (RAEB), which can often transform into acute leukemia. Although the association between HM and AAV is thought to be rare, its early recognition is relevant for several reasons. To begin with, missing the neoplastic trigger in a patient presenting with an AAV may have catastrophic consequences for his prognosis. Moreover, even when promptly spotted, patients who suffer from both diseases seem to face a much worse prognosis than that expected for both groups of diseases (i.e., AAVs and hemopathies) when presenting independently. Secondly, the high infectious risk, probably heightened by the synergy of vasculitis, hemopathy, and the treatment favouring immune dysfunction, seems to be a major complication for those patients [21].This sparks interest in strategies trying to minimize treatments [20]. Another aspect to consider regards the differential diagnosis of both entities; in fact, lymphoma has been reported to present in a way that mimics vasculitis [22, 23] and the opposite clinical picture has also been widely described in scientific literature [24]. Lastly, the incidence of vasculitis occurring in close temporal association with cancer cannot be overemphasized, with some studies suggesting that these cases may constitute from 0.4 to 4.2% of all vasculitis cases [25].

## 3. Increased Risk of Hemopathies in AVV: Is It Just the Effect of Treatment?

Most studies which investigated the relationship between AAV and leukemias reported an increased risk of developing some sort of blood neoplasm in those patients who underwent immunosuppressive treatment, especially with cyclophosphamide [26] (Table 1). In 2015, Shang et al. performed the first meta-analysis on this topic, and analysing previous studies (pooled leukemia standardized incidence ratio (SIR) of 4.89 (95%CI = 2.93–8.16)) revealed a significant association with the use of this drug [27]. Consequently, they recommended that all AAV patients previously treated with cyclophosphamide should undergo long-term follow-up with routine blood examination for early detection of leukemia [27]. There is little doubt that this effect is largely mediated by the direct oncogenic effect of cyclophosphamide [25], which, together with glucocorticoids and other agents, has long been one of the main immunosuppressant drugs used in AAV. Cyclophosphamide is known to be associated with an increased risk of leukemia in a dose-dependent manner [25, 28, 29]. To prevent the daunting side effects of the drug, including increased risk of urothelial tumours and nonmelanoma skin cancer, two recent clinical trials investigated the use of rituximab, an anti-CD20 monoclonal antibody, as an alternative induction agent. In 2010, Stone et al. published the results of the RAVE trial (a multicenter, randomized, double-blind, noninferiority trial comprising 197 patients) at a 6-month endpoint: the rituximab-based protocol was found to be more effective and did not differ in safety from the cyclophosphamide one [30]. A similar trial was published thereafter on the same topic with results which were not completely overlapping; in fact, the RITUXVAS trial (an open-label, two-group, parallel-design, randomized trial involving 44 patients) failed to show rituximab superiority in terms of either efficacy or safety following one year. However, rituximab did not result as significantly inferior either in this smaller group of patients [31]. The authors also provided updated information about the outcome of the RITUXVAS trial after 24 months, with results similar to the previous ones [32]. In 2017, van Daalen et al. directly compared the risk of malignancy in AAV patients treated with cyclophosphamide and those treated with rituximab [33]. They included in their study 323 patients for a mean follow-up of 5.6 years. The overall risk for all malignancies was statistically increased in the whole cohort (SIR, 1.89; 95% CI 1.38 to 2.53). This did not apply to hematologic malignancies; however, patients treated with cyclophosphamide had a risk of developing cancer significantly higher than the general population (SIR, 3.10; 95% CI 2.06 to 4.48) and manifold higher in patients treated with rituximab (SIR, 4.61; 95% CI 1.16 to 39.98) [33]. These findings seem to support the hypothesis that cyclophosphamide is accountable for most of the increased incidences of malignancy in AAV patients. At first glance, adopting rituximab instead of cyclophosphamide in all patients may be the definitive solution. However, the possibility exists that a combination regimen of corticosteroids, cyclophosphamide, and rituximab will prove to be more effective than single-agent therapy. This is suggested by a recent case control study including 66 patients for a median follow-up of 4.6 years [34]. In this study, a low-dose intravenous pulsed regimen of cyclophosphamide was used: this strategy may diminish the incidence of dose-dependent adverse reactions without having to give up altogether on the use of such an effective agent as cyclophosphamide. Moreover, the efficacy and toxicity of rituximab, cyclophosphamide, and combined regimens will need to be investigated on larger cohorts of patients and for longer follow-up periods in order to get a definitive picture of the most convenient treatment choice. Studies which include few patients or consider brief periods of time may be underpowered to show a statistically significant effect on cancer incidence, especially single-cause incidence, and therefore are probably of no use in this specific issue. Meanwhile, the possibility of intrinsic mechanisms leading to increased cancer risk, which could be linked to the pathogenesis of ANCA-associated vasculitis, should not be overlooked by concerns with the well-known cyclophosphamide toxicity [27]. Several autoimmune disorders are characterized by long-standing self-directed immune responses implicated in lymphomagenesis, especially non-Hodgkin's lymphoma (NHL) [35]. The strongest evidence refers to Sjogren's syndrome (SS), where independent risk factors such as enlargement of salivary glands, lymphadenopathy, Raynaud phenomenon, anti-SSA or/and anti-SSB positivity, RF positivity, and monoclonal gammopathy were all found to be significantly associated with NHL development [36, 37]. Of course, none of these factors play any relevant role in AAV. Still, it would be of interest to evaluate whether features such as cANCA and/or pANCA, multiple organ involvement, response to treatment, and decades of presentation may be significant in increasing HM risk. Unfortunately, stratifying AAV patients into subcohorts is a challenging task made difficult by the low incidence of these diseases, which are reported to occur in less than 10 per million patients every year by most studies [38]. Moreover, the difficulty of data collection in AAV is strikingly clear if compared to the overall incidence rate of primary SS reported by a recent meta-analysis as tenfold higher [39]. Another possible culprit of oncogenesis in autoimmune disorders is the presence of chronic tissue inflammation. In this case, an inflammatory microenvironment is responsible for the generation of reactive oxygen species and free radicals which over time may induce genetic changes and ultimately the onset of a neoplasia as in the well-known association between ulcerative colitis and colorectal cancer [40]. As far as the HM are considered, chronic lymphocytic leukemia (CLL) has been found to be dependent upon an inflammatory microenvironment and increased inflammatory cytokine signalling when cultured in vitro [41]. Although associations between AAV and CLL have been reported in the literature [42–44], they seem to fit better into the group of paraneoplastic rather than underlying AAV. At present, no significant role can be attributed to AAV vessel inflammation in stimulating oncogenesis. Wester Trejo et al. extensively focused their attention on other mechanisms that might increase cancer risk in AAV patients and summarized those in a comprehensive review [45]. The authors found contradictory evidence regarding a possible shared pathogenesis and concluded that suspicion of a neoplastic process should not be increased in AAV patients [45]. Another study published in 2018 by Yoo et al. found similar results; indeed, the analysis performed on a cohort of Korean patients was not statistically significant with respect to an increased risk of cancer overall or in any subgroups [46]. In such a complex scenario, a wide multicenter effort would seem fundamental to obtain enough and long-term data on rituximab-treated patients that could, through their careful interpretation, verify the possibility of an AAV-related oncogenic mechanism. This is especially true when considering the theoretical ambivalence of PR3 function in GPA and acute myeloid leukemia, which is the focus of the next paragraph.

## 4. Proteinase 3: A Molecule at the Crosstalk of Autoimmunity and Hematopoietic Proliferation

Anti-neutrophil cytoplasmic antibodies are autoantibodies capable of causing systemic vascular inflammation by binding to their target antigens, mostly expressed by polymorphonuclear cells (PMN) [21, 47]. They are routinely used as biomarkers in AAV and are detected by both serum indirect immunofluorescence (IIF) studies and ELISA [26, 48]. Positive ANCA can be classified into three categories based on their IIF pattern: cytoplasmic (cANCA), perinuclear (pANCA), and atypical (aANCA) [49]. cANCA are usually associated with GPA, pANCA are mostly found in MPA and less frequently in EGPA, while aANCA are associated with levamisole-contaminated cocaine exposure in abusers and other conditions [21, 50]. The cANCA pattern is mostly due to the presence of anti-PR3 autoantibodies while the pANCA pattern is usually due to the presence of anti-myeloperoxidase (anti-MPO) antibodies. The latter molecule is a cationic enzyme present in azurophilic granules of neutrophils with a strong bactericidal activity [21]; instead, PR3 is a serine protease homologue found within neutrophils and monocytes granules [51–53]. Regarding PR3, it is important to note that along with being GPA's main autoantigen, it is also overexpressed in several HM such as acute and chronic myeloid leukemia cell lines [54, 55]. This unique situation may be explained by the complex array of physiological and possibly pathophysiological cell functions of PR3 [56]. In 2001, van der Geld et al. identified three major areas which needed to be addressed in order to better understand the function of PR3: its genetic localization and gene regulation, the processing and storage of the protein, and its physiological function. PR3 expression was found to be limited to cells of granulocytic and monocytic lineages, being localized in the granules of mature monocytes and neutrophils. Moreover, PR3 expression was also observed on the membrane of resting neutrophils, with increasing amounts in patients with active GPA [56]. This bimodal distribution seems to be a unique feature of PR3. While endothelial expression of PR3 would have nicely explained some features in the pathogenesis of GPA and although some interactions between PR3 and endothelial receptors were found [56], further experiments are still required for confirmation. PR3 expression is upregulated in hematopoietic progenitor cells under the stimulus of granulocyte colony-stimulating factor (G-CSF) [56] and is believed to enhance the proliferation rate of those cell lines in the bone marrow [56]. Moreover, the downregulation of this molecule induces differentiation of granulocyte precursors, and, in contrast, the loss of control on this metabolic pathway may favour the development of myeloid leukemia [56]. Other biologic roles in which PR3 is involved refer to the destruction of phagocytosed microbes, active involvement in diapedesis, modulation of inflammation, and even induction of endothelial apoptosis [56]. The main inhibitor of PR3 was found to be alpha-1-antitrypsin (*α*_1_-AT). This fits well with the finding that some patients with abnormal *α*_1_-AT gene alleles have increased risk of GPA and other disorders (mostly liver disease and emphysema, depending on the mutations involved) [56].

**Figure 1:**
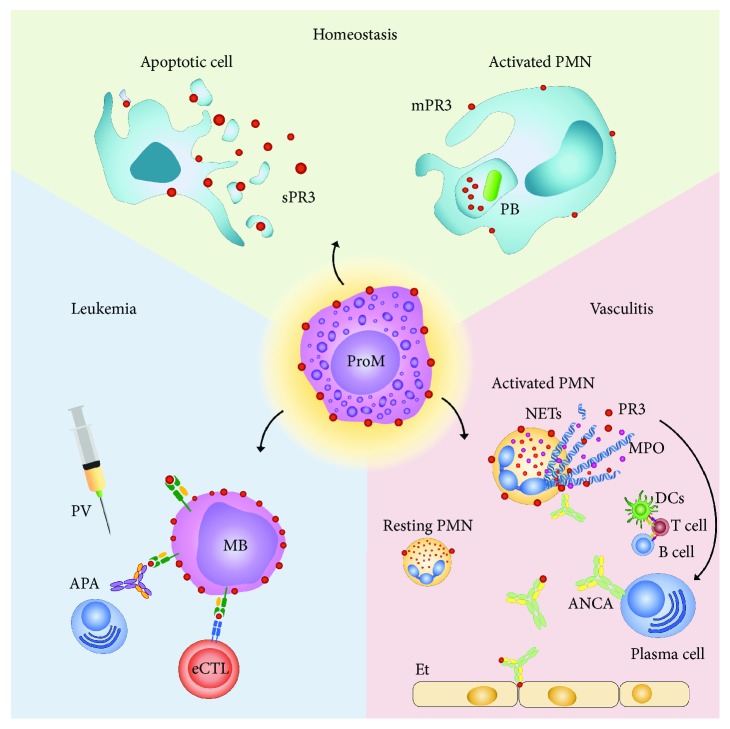
PR3 biological roles in healthy status and diseases. Center (yellow): the myeloid precursor, promyelocyte, expresses a great amount of PR3 on its surface under G-SCF stimulus, and it is believed that PR3 is able to enhance the proliferation rate of those cell lines in the bone marrow. Homeostasis (green): PR3 allows destruction of phagocytosed bacteria in activated polymorphonuclear cells; moreover, PR3 can interact with apoptotic cells and microvesicle binding phosphatidylserine on the cell surface. PR3 favours diapedesis and works as a modulator of inflammation. AVV (red): PR3 is the main autoantigen in GPA and MPA. It may favour inflammation by interfering with apoptosis or leading to endothelial damage because of ANCA generation that is proved to be enhanced under leukocyte activation, especially neutrophil NETosis. Leukemia (blue): PR3 does not downregulate in leukemia. PR1-specific CTLs and anti-PR1-HLA-A2 are being developed against AML blasts. A PR1 peptide vaccination has already been experimented with success in humans. Abb.: APA: anti-PR1/HLA-A2 antibodies; PV: PR1 peptide vaccination; eCTL: cytotoxic lymphocyte; MB: myeloid blast; PR1/HLA-A2: PR1 peptide being presented by HLA-A2; mPR3: membrane proteinase 3; PB: phagocytosed bacterium; sPR3: soluble proteinase 3; ProM: promyelocyte; resting PMN: resting polymorphonuclear cell; activated PMN: activated polymorphonuclear cell; ANCA: anti-neutrophil cytoplasmic antibodies; Et: endothelium; NETs: neutrophil extracellular traps.

Given the extensive knowledge on PR3 mechanisms of function, it is puzzling that its pathogenicity in GPA has not yet been proven beyond reasonable doubt [11]. While anti-MPO IgG pathogenicity has been established in murine models, the same does not apply to PR3 ANCA [57]. Attempts to reach a definite conclusion may have been unsuccessful due to differences in PR3 structure between humans and other animals as well as differences concerning its intracellular storage and processing [11]. The presence of a hydrophobic patch in human PR3 seems to facilitate its association with various lipids and proteins and permits its anchorage to the plasma membrane [11]. Moreover, the ability of PR3 to bind phosphatidylserine (PS) may allow it to interact with apoptotic cells displaying this phospholipid on their cell surface and microvesicles [11]. Even soluble PR3 may bind to PS on the cell surface (especially apoptotic neutrophils, macrophages, and microvesicles) and remain bound to bystander cells. This could prevent the physiological clearance of these cells due to PR3 interference with the apoptotic process, generating a source of autoantigens [11]. Another major implication of PR3 function in pathophysiology concerns its ability to suppress in vitro T cell-mediated immunity in AML [58]; in fact, clinical studies have shown a worse clinical outcome when PR3 is present in the tumour microenvironment which can partially be derived from the potentiation of tumour angiogenic properties [58]. The role of this molecule in suppressing adaptive immunity is fascinating, although probably dependent upon the features of the microenvironment in which it is present. It may be even hypothesized that PR3 binding to cANCA in GPA prevents it from exerting its tolerogenic action and therefore favours autoimmunity [58], even if this theory is somewhat opposite to the previously described role of PR3 as a proinflammatory molecule [11]. All things considered, PR3 depiction seems to remind that of a tightrope walker trying to avoid an equally catastrophic fall on both sides of the rope. However, it is important to consider how this interpretation could be limited by a lack of full understanding of the biological contexts in which PR3 is involved (Figure 1). This may imply that PR3 can be driven towards different directions by additional agents, thus acquiring opposite effects.

As mentioned in the introduction, proteasome processing of PR3 leads to the production of a nine-amino acid peptide, PR1, which is presented by HLA A2. PR1 has been studied as an immunotherapy target in AML, with the development of genetically modified T cells [5], anti-PR1/HLA-A2 antibodies [8], and a PR1 peptide vaccination [6]. Among these, only the peptide vaccination is currently in clinical phase of development. A phase I/II clinical trial showed safety and efficacy of the vaccine in 66 patients with AML [6, 59]. Interestingly, none of the patients developed cANCA nor clinical manifestations of vasculitis [6]. This was comprehensibly one of the researchers' safety endpoints, provided that PR1 is expressed by normal hematopoietic cells alike, although at lower levels [8, 59]. The same research group, who developed the PR1 vaccine, recently extended its focus to PR1 targeting in multiple myeloma [60]. While myeloma cells do not show endogenous expression of PR3, they were found to uptake exogenous soluble PR3, process it through the proteasome, and cross-present it on the cell surface by HLA-A2 [60]. Since myeloma cells originate from B cells, which are APCs themselves, this mechanism seems to have been retained through neoplastic transformation [60]. This implies that myeloma does not belong to those tumours downregulating their MHC class I molecules to evade immune control [61]. Moreover, PR1 cross-presenting myeloma cells were found to be susceptible to both CTLs and anti-PR1/HLA-A2-mediated killing, and although data were still preliminary, they let PR1 emerge as a promising new prospect in multiple myeloma [60].

## 5. Conclusions

It will still take time to extend treatment to larger experimental cohorts and evaluate whether a clinical benefit with respect to existing therapies could be provided to AML and MM patients. It is certain, however, that the novel therapeutic approaches being developed in the field of leukemia, based on targeting PR3, will be followed with interest by all clinicians interested in understanding the pathogenesis of AAV and the specific role of PR3 in the development of GPA.

## Figures and Tables

**Table 1:** Studies investigating the incidence of leukemia and all hematopoietic tumours in AAVs.

	Knight et al. 2002 [62]	Faurschou et al. 2008 [63]	Holle et al. 2011 [64]	Heijl et al. 2011 [65]	Zycinska et al. 2013 [66]	Faurschou et al. 2015 [67]	Rahmattulla et al. 2015 [68]	van Daalen et al. 2017 [33]	Sriskandarajah et al. 2017 [69]	Heaf et al. 2018 [70]	Yoo et al. 2018 [46]
SIR of leukemia (95% CI)	5.7 (2.3–12)	5.9 (1.2-17)	Not statistically increased	3.2 (0.4-11.7)	4.3 (2.1-11.2)	13.3 (3.6-34)	Not Statistically increased	Not statistically increased	—		Not statistically increased
Observed minus expected leukemia cases (%) if significant	—	—	—	—	—	—	—	—	—	11	—
SIR of all hematopoietic tumours (95% CI)	3.8 (2.1–6.3)	—	—	—	—	1.9 (0.5–5.0)	Not statistically increased	Not statistically increased	3.52 (1.32–9.37)	—	Not statistically increased
Study period	1969-1994	1973-1999	1966-2002	1995-2002	1990-2008	1973-1999	1991-2013	2000-2014	1988-2012	1985-2015	—
Patient number	1065	293	290	535	117	293	138	323	419	278	150
Patient composition	GPA only	GPA only	GPA only	AAV	AAV	GPA only	AAV	AAV	AAV	AAV	AAV
Country	Sweden	Denmark	Germany	Europe, Mexico	Poland	Denmark	Netherlands	Europe	Norway	Denmark	Korea
Data collection	National study	National study	Single-center study	Multicenter study	Single-center study	National study	Single-center study	Single-center study	National study	National study	Single-center study
